# Thoracoscopic Pulmonary Vein and Left Atrial Posterior Wall Isolation Combined with Left Atrial Appendage Resection in Patients with Long-Standing Persistent Atrial Fibrillation

**DOI:** 10.21470/1678-9741-2019-0132

**Published:** 2020

**Authors:** Aleksandr Zotov, Sergei Vachev, Daniil Borisov, Aleksandr Troitskiy, Robert Khabazov

**Affiliations:** 1 Department of Cardiac Surgery, Federal Research and Clinical Centre, Moscow, Russian Federation.

**Keywords:** Atrial Fibrillation, Pulmonary Veins, Ischemic Attack, Transient, Sternotomy, Thoracotomy, Myocardial Infarction, Postoperative Complications

## Abstract

**Objective:**

To evaluate the efficacy and safety of a modified technique for totally thoracoscopic left atrial posterior wall and pulmonary vein isolation in patients with long-standing persistent atrial fibrillation.

**Methods:**

From April 2017 to December 2018, we included in this study 28 consecutive patients who underwent thoracoscopic left atrial posterior wall and pulmonary vein radiofrequency isolation combined with left atrial appendage resection. We used a device with irrigated electrodes (Medtronic Cardioblate Gemini-s). The original surgical technique “GALAXY” proposed by Doty in 2012 was modified. The number of ablations was significantly increased, and frequent position changing of the ablation device and change of device angulation were added.

**Results:**

Sinus rhythm was restored in all patients. There was no operative mortality, no myocardial infarction, and no stroke or transient ischemic attack. One patient required sternotomy and another survived left anterolateral thoracotomy due to bleeding. A 180-day follow-up (24-hour Holter monitoring) revealed no sign of recurrence of atrial fibrillation or other supraventricular arrhythmia in any patient. Mean follow-up was nine months (range: 6-16 months). At the last follow-up, 26 patients (92,9%) were in sinus rhythm (24-hour Holter monitoring).

**Conclusion:**

A frequent ablation device position changing during the surgery makes it possible to achieve complete left atrial posterior wall and pulmonary veins isolation.  An increased number of applications allows to avoid a false positive transmural damage assessment showed by impedance drop. Also, frequent position changing of the ablation device and increased number of applications do not affect the number of postoperative complications.

**Table:** 

Abbreviations, acronyms & symbols	
AF	= Atrial fibrillation
CM III	= Cox-Maze III
LA	= Left atrium
pts	= Patients
PV	= Pulmonary veins
RFA	= Radiofrequency ablation

## INTRODUCTION

Atrial fibrillation (AF) has a rising prevalence in the general population, and this condition is associated with reduced long-term survival and impaired quality of life^[1]^. The low quality of life is caused by need for constant anticoagulation, antiarrhythmic, and other negative chronotropic drugs. These can cause significant reduction in functional capacity and development of significant complications with time^[2-5]^.

Thromboembolism is the most serious complication of AF. Thrombi can form in the absence of contractility of the atria and the left atrial appendage, with potential migration of these blood clots into the distal arteries (embolism), hence necessitating long-term anticoagulation^[6]^. Pertinent negatives of anticoagulation include the constant risk of bleeding, possible restriction of certain physical activities, difficulty in controlling the effectiveness of warfarin, and associated costs^[7-9]^. Long-term persistent AF can lead to occurrence and subsequent decompensation of congestive heart failure^[2,3,10]^. AF is generally classified into paroxysmal (duration up to seven days with spontaneous termination), persistent (lasts more than seven days and requires medical or electrical cardioversion to eliminate it), long-standing persistent (duration ≥ 1 year), and permanent (when the patient and the doctor determine that long-term maintenance of sinus rhythm is not an option)^[4,5]^.

Rhythm control for AF is indicated for symptomatic patients^[4,5]^. Long-term success rates of catheter ablation (single and multiple) are low, especially in persistent and long-standing persistent AF.

Evolution of surgical treatment of AF has led to the emergence of various techniques of thoracoscopic epicardial exposure of the left atrium (LA) and subsequent pulmonary veins (PV) and posterior wall isolation. Such operations are attractive in having lower morbidity than the "gold standard" Cox-Maze III (CM III) procedure, but approach it in efficiency^[8,10,11]^. The most modern thoracoscopic epicardial surgical techniques include radiofrequency ablation using bipolar ablation devices to isolate PV and the entire posterior wall of the LA^[12]^. The additional benefits of posterior wall isolation include elimination of AF triggers and interruption of re-entry loops that are felt to be responsible for the maintenance of AF^[13]^.

This study is designed to evaluate the immediate and medium-time efficacy and safety of thoracoscopic ablation for long-standing persistent AF using the Medtronic Cardioblate Gemini-s ablation device (Medtronic, Minneapolis, Minnesota, USA).

## METHODS

### Patients’ Characteristics

From April 2017 to December 2018, we included in this study 28 consecutive patients who underwent radiofrequent epicardial left atrial posterior wall and PV isolation for long-standing persistent AF using thoracoscopic approach. Clinical and demographic characteristics of those patients are in Table 1. There were no patients with symptomatic sinus node dysfunction in our group. Two patients included in this study had prior endocardial cavo-tricuspid isthmus dependent atrial flutter ablation.

**Table 1:** Clinical and demographic characteristics of the patients (n=28).

Male, number of pts (N [%])	21 [75%]
Age, years (median [min-max])	60 [42-73]
Body mass index (median [min-max])	29 [23-40]
Duration of atrial fibrillation, months (median [min-max])	113 [12-485]
History of thromboembolic complications, number of pts (N [%])	3 [10.7%]
Stroke	2
Pulmonary embolism	1
Indexed left atrium volume, ml/m^2^ (median [min-max])	42 [28-57]
Left ventricular ejection fraction, Simpson, % (median [min-max])	55 [38-66]
Pulmonary artery pressure, mmHg (median [min-max])	32 [26-49]
Hypertension, number of pts (N [%])	25 [89.3%]
Drug-refractory tachycardia (> 90/min), number of pts (N [%])	11 [39.3%]
Endocardial RFA of pulmonary veins in the past, number of pts (N [%])	3 [10.7%]
Endocardial RFA of cava-tricuspid isthmus in the past, number of pts (N [%])	2 [7.1%]

pts=patients; RFA=radiofrequency ablation

### Surgical Technique

To perform the surgical intervention, we used the Cardioblate Gemini-s ablation device (Medtronic, Minneapolis, Minnesota, USA) with irrigated electrodes. Basic surgical technique was as recommended by the manufacturer and described by Doty et al.^[14]^ in original manuscript^[12,14,15]^. Briefly, the surgery is performed through three thoracic ports with pericardial reflection and sequential single lung ventilation. Soft grey and blue rubber catheters are passed behind the LA, above and below the superior and inferior PV, respectively, to guide the device advancement while avoiding trauma.

The curved device is first inserted into the pericardial cavity from the left side, guided by the soft rubber catheters, up to the patient's spine^[14]^. Once appropriately positioned, it is clamped over the left half of the LA. Three lesions are applied guided by impedance drop to confirm transmurality. After each application, the device is adjusted a little to get overlapping lesions. The process is repeated on the right side to obtain isolation of PV as well as a large portion of the posterior LA wall – hereby creating a "box lesion".

**Fig. 1:**
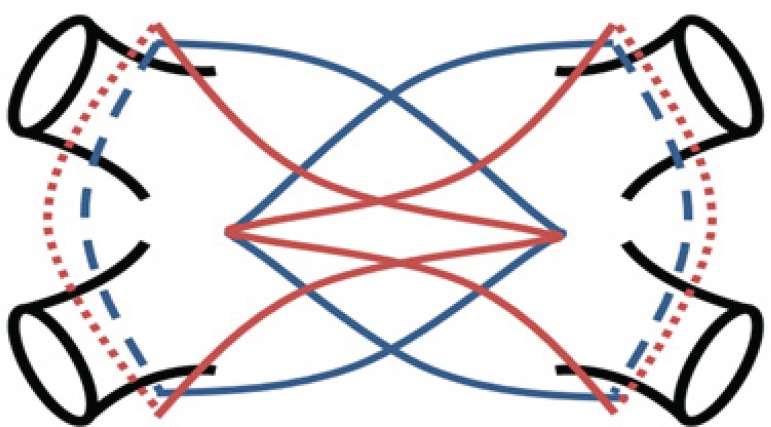
Scheme of the left atrial ablation lines.

**Fig. 2:**
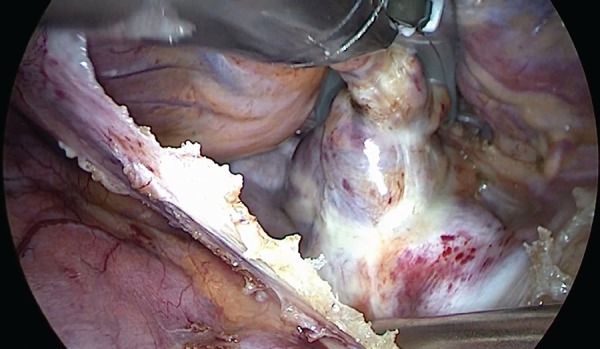
Left atrial posterior wall ablation.

At our institution, to obtain transmurality, we initially perform 10 lesions from each side with 3-5 mm adjustment between ablations. The device is then removed, cleaned, and the char removed; the same side is repeated with a downward angulation of the device for 10 more lesions, totalling 20 lesions on each side (Figures 1 and 2).

The left atrial appendage is resected with a Medtronic Echelon (Medtronic, Minneapolis, Minnesota, USA) stapler and a "60+" cassette.

This technique with increased number of applications helps to reach complete and permanent PV and left atrial posterior wall isolation and to destroy the ligament of Marshall.

Finally, bilateral epicardial stimulation is performed to check the block of conduction.

Cardiac output monitoring is not needed during this procedure because invasive arterial blood pressure monitoring provides enough information to evaluate hemodynamics. We don’t count incomplete clampings of the veins in order to achieve full transmurality. Sometimes prolonged intervals between clampings with subsequent position changing are needed due to hypotension. The total number of complete clampings is always 40.

### Statistical Data Analysis

Microsoft^®^ Excel 2010 was used for statistical data analysis. The data was presented as median and range or number and percentage.

## RESULTS

A total of 28 patients were included in our study.

All patients were discharged with antiarrhythmic therapy in order to maintain sinus rhythm (amiodarone, 200 mg daily), beta-blockers (individual dosages), anticoagulants (warfarin), and antihypertensive drugs (if necessary).

Twenty-eight patients (100%) underwent a control examination 90 and 180 days after the procedure. Chest X-ray and 24-hour Holter monitoring were evaluated.

Postoperative results (Table 2) were studied in 28 patients (100%). The median surgery time was 137 (118-193) minutes. The median ablation time was 29 (23-42) minutes.

**Table 2:** Postoperative results (n=28).

Surgery time, minutes (median [min-max])	137 [118-193]
Ablation time, minutes (median [min-max])	29 [23-42]
Intubation time, hours (median [min-max])	8 [3-36]
Time with chest tubes, hours (median [min-max])	28 [16-74]
Intensive care unit stay, days (median [min-max])	1 [0.6-8]
Hospital stay, days (median [min-max])	5 [3-13]
Postoperative complications	
Need for blood product transfusion, number of pts (N [%])	0
Incidence of pneumonia, number of pts (N [%])	0
Need for reintubation, number of pts (N [%])	0
Unilateral phrenic nerve palsy, number of pts (N [%])	2 [7.1%]
Transient bilateral phrenic nerve palsy with prolonged non-invasive ventilation, number of pts (N [%])	1 [3.6%]

pts=patients

At the end of surgery, the block of conduction was achieved and confirmed by subsequent stimulation in 100% of patients.

Sinus rhythm was restored in all patients. Electrical cardioversion on the operating table was effective in 15 patients. In 13 patients, sinus rhythm was restored 24 hours later, after amiodarone and potassium chloride infusion and electrical cardioversion.

There was no operative mortality, no myocardial infarction, and no stroke or transient ischemic attack. One patient required sternotomy and another survived left anterolateral thoracotomy due to perforation of the superior vena cava and bleeding; none of them required cardiopulmonary bypass connection. The defect was sewn with Prolene 4-0 in both cases. One patient required prolonged non-invasive ventilation for transient bilateral phrenic nerve palsy with complete recovery confirmed by fluoroscopy 10 days after the procedure. Right phrenic nerve injury was documented in two patients, but none of them required prolonged ventilation.

Overall safety profile (intraoperative and early complication rates) suffered from a steep learning curve. This is dependent not only on the initial experience of the surgeon but also on the surgeon’s ability to organize a systematic approach to this operation.

Anticoagulation therapy, beta-blockers, and amiodarone were cancelled for all patients 90 days after the surgery. Seven days later, all patients routinely underwent follow-up examination, which included chest X-ray and 24-hour Holter monitoring. A total of 28 patients (100%) were examined. Two patients with right phrenic nerve injury (7.1%) had restricted mobility of the right hemidiaphragm dome. However, dyspnea or recurrent pulmonary infections have not been documented in these patients. So, we can conclude that this complication did not have significant impact on the patients’ postoperative symptoms.

After a 90-day follow-up, beta-blockers were the only medicines continued in all patients.

A 180-day follow-up (24-hour Holter monitoring) revealed no sign of recurrence of AF or other supraventricular arrhythmia in any patient.

Mean follow-up was nine months (range: 6-16 months). At the last follow-up, 26 patients (92.9%) were in sinus rhythm, as documented by 24-hour Holter monitoring.

AF recurrence occurred in two patients. However, the postoperative form of AF in these patients could be described as paroxysmal. Therefore, the procedure transformed the long-standing persistent AF into paroxysmal AF.

## DISCUSSION

Each surgical technique should be safe for patients, efficient, and easy for learning and introduction. In most cases, these conditions are mutually exclusive. Safety could be reached by minimizing the surgical trauma and shortening the incision. On the other hand, decreased trauma may affect efficiency, so the whole surgical procedure becomes irrelevant.

The most effective technique for surgical treatment of AF is the CM III procedure. It became the gold standard in surgical treatment of AF. However, due to technical complexity (median sternotomy and the use of a cardiopulmonary bypass) and assumed increase in morbidity, CM III is not widely used nowadays^[16,17]^.

Development of radiofrequency and cryoablation devices reduced the risks and trauma of the CM III procedure (fewer incisions on the heart were needed)^[18]^. Right thoracotomy approach was proposed in order to decrease the invasiveness of the procedure, but it still required cardiopulmonary bypass use^[19]^. Afterwards, it was discovered that the left atrial posterior wall and the PV are a dominant source of recurrent rapid activity emanating continuously, intermittently, or alternately during AF^[20,21]^. Since then, it became apparent that the most important part of the CM III procedure is the left atrial posterior wall and PV isolation (box lesion)^[9,21,22]^. Subsequent development of radiofrequency devices and introduction of thoracoscopic approach provided further minimization of invasiveness and made this procedure feasible without cardiopulmonary bypass^[12,23,24]^.

According to recent studies^[25]^, the most appropriate devices for beating heart PV isolation are bipolar radiofrequency clamps, but testing for exit or entrance block is mandatory. Surface bipolar radiofrequency devices may be recommended for free wall linear ablation when lesion integrity can be tested, however multiple applications are recommended to achieve adequate lesion depth, which requires additional time. Epicardial cryoablation is not recommended for beating heart surgery, and there are no surgical devices for thoracoscopic cryoablation approach nowadays.

Thus, the modern formula for surgical AF treatment rejects the use of cardiopulmonary bypass and includes thoracoscopic approach and the use of bipolar clamps for epicardial radiofrequency ablation in order to isolate the left atrial posterior wall and PV. According to previous studies, bipolar radiofrequency ablation is able to achieve transmural damage in most cases and shows promising long-term results^[17,26]^.

The Cardioblate Gemini-s ablation device (Medtronic, Minneapolis, Minnesota, USA) fits this formula. An original technique is called “GALAXY” and implies thoracoscopic left atrial posterior wall and PV isolation combined with the left atrial appendage resection and the ligament of Marshall destruction^[14]^. The device is inserted into the pericardium through a thoracic port, guided by the catheters. Once appropriately positioned, it is clamped over the half of the LA. From one to three lesions are applied guided by impedance drop to confirm transmurality. This algorithm is based on determining tissue resistance and sometimes it gives false positive results^[26]^. That’s why we use the modified protocol described above. This modified technique allowed us to achieve a complete block of conduction in all patients included in this study, which was confirmed by intraoperative high frequency epicardial stimulation.

Additionally, the modified technique shows promising medium-term results (all patients were in sinus rhythm 180 days after the surgery), taking into account that all patients had a long history of persistent AF before the procedure (median: 23 [18-36] months).

## CONCLUSION

A frequent position changing of the ablation device during the surgery makes it possible to achieve complete left atrial posterior wall and PV isolation. An increased number of applications allows to avoid a false positive transmural damage assessment showed by impedance drop. Also, frequent position changing of the ablation device and increased number of applications do not affect the number of postoperative complications.

**Table:** 

Author's roles & responsibilities	
AZ	Substantial contributions to the conception or design of the work; or the acquisition, analysis, or interpretation of data for the work; drafting the work or revising it critically for important intellectual content; agreement to be accountable for all aspects of the work in ensuring that questions related to the accuracy or integrity of any part of the work are appropriately investigated and resolved; final approval of the version to be published
SV	Substantial contributions to the conception or design of the work; or the acquisition, analysis, or interpretation of data for the work; drafting the work or revising it critically for important intellectual content; agreement to be accountable for all aspects of the work in ensuring that questions related to the accuracy or integrity of any part of the work are appropriately investigated and resolved; final approval of the version to be published
DB	Substantial contributions to the conception or design of the work; or the acquisition, analysis, or interpretation of data for the work; drafting the work or revising it critically for important intellectual content; agreement to be accountable for all aspects of the work in ensuring that questions related to the accuracy or integrity of any part of the work are appropriately investigated and resolved; final approval of the version to be published
AT	Substantial contributions to the conception or design of the work; or the acquisition, analysis, or interpretation of data for the work; drafting the work or revising it critically for important intellectual content; agreement to be accountable for all aspects of the work in ensuring that questions related to the accuracy or integrity of any part of the work are appropriately investigated and resolved; final approval of the version to be published
RK	Substantial contributions to the conception or design of the work; or the acquisition, analysis, or interpretation of data for the work; drafting the work or revising it critically for important intellectual content; agreement to be accountable for all aspects of the work in ensuring that questions related to the accuracy or integrity of any part of the work are appropriately investigated and resolved; final approval of the version to be published
